# Application of Three-Dimensional Imaging to the Intestinal Crypt Organoids and Biopsied Intestinal Tissues

**DOI:** 10.1155/2013/624342

**Published:** 2013-11-14

**Authors:** Yun Chen, Ya-Hui Tsai, Yuan-An Liu, Shih-Hua Lee, Sheng-Hong Tseng, Shiue-Cheng Tang

**Affiliations:** ^1^Department of Surgery, Far Eastern Memorial Hospital, Pan-Chiao, New Taipei 220, Taiwan; ^2^Department of Chemical Engineering and Materials Science, Yuan Ze University, Chung-Li, Taoyuan 320, Taiwan; ^3^Department of Chemical Engineering, National Tsing Hua University, Hsinchu 300, Taiwan; ^4^Department of Surgery, National Taiwan University Hospital, National Taiwan University College of Medicine, Taipei 100, Taiwan; ^5^Department of Medical Science, Institute of Biotechnology, National Tsing Hua University, Hsinchu 30013, Taiwan

## Abstract

Two-dimensional (2D) histopathology is the standard analytical method for intestinal biopsied tissues; however, the role of 3-dimensional (3D) imaging system in the analysis of the intestinal tissues is unclear. The 3D structure of the crypt organoids from the intestinal stem cell culture and intestinal tissues from the donors and recipients after intestinal transplantation was observed using a 3D imaging system and compared with 2D histopathology and immunohistochemistry. The crypt organoids and intestinal tissues showed well-defined 3D structures. The 3D images of the intestinal tissues with acute rejection revealed absence of villi and few crypts, which were consistent with the histopathological features. In the intestinal transplant for megacystis microcolon intestinal hypoperistalsis syndrome, the donor's intestinal tissues had well-developed nerve networks and interstitial cells of Cajal (ICCs) in the muscle layer, while the recipient's intestinal tissues had distorted nerve network and the ICCs were few and sparsely distributed, relative to those of the donor. The 3D images showed a clear spatial relationship between the microstructures of the small bowel and the features of graft rejection. In conclusion, integration of the 3D imaging and 2D histopathology provided a global view of the intestinal tissues from the transplant patients.

## 1. Introduction

Histopathological analysis of intestinal biopsies provides important information for the evaluation and diagnosis of intestinal diseases and is used in the diagnosis of graft rejection after intestinal transplantation. This conventional 2-dimensional (2D) method is fast, straightforward, and inexpensive and is a routine procedure in practice. Although 2D histology is the standard method for tissue analysis, diagnostic errors and inconclusive results are inevitable, as only a few sections of the specimen are from the bulk tissue [1–6]. Exhaustive sectioning and the incorporation of each slice into a 3D-like virtual structure had been proposed to solve this problem; however, this method is time-consuming and costly [7]. Recently, a 3D imaging system that combines the optical clearing method, fluorescence staining, and the confocal microscope has been developed [6, 8]. The standard 2D tissue analysis confines the visualization of the intestinal architecture at a specifically cut plane; however, the 3D images represent a complete area of interest [6]. Importantly, because the tissues are thick and opaque, an optical clearing agent is necessary to increase the transparency and reduce the light scattering when preparing the 3D images [9]. Next, the tissues are subjected to optical imaging, laser penetration, and fluorescence detection by confocal microscopy [8]. Confocal microscopy is widely used in biomedical investigations for visualizing the internal structure of biological tissues [10]. It generates sharp 2D images at the plane of focus; incrementing the plane of focus creates a series of optical sections at different depths in the specimen, which allows for construction of a 3D image [11]. From these images, the deep structures of the regions of interest in the whole-mount tissues can be visualized [7, 12]. This method is fast, straightforward, and inexpensive, as compared to 3D image reconstruction from histological slices [12].

The intestine is comprised of the mucosa, submucosa, muscle layers, and serosa, as well as a proliferating stem cell population located at or near the crypt base of the mucosa, which undergoes terminal differentiation as these cells migrate to the luminal surface [13]. Because *in vitro* cultures of intestinal stem cells (ISCs) have been shown to develop into crypt organoids, which contain all differentiated cell types of the villi, and the ISCs may develop into neointestinal mucosa [14–20], it is interesting to observe the 3D structures of the crypt organoids. Graft rejection after intestinal transplantation is usually diagnosed according to the histopathology of biopsied intestinal tissues, including the changes of the lamina propria, crypts, villi, and mucosa, in addition to the mononuclear cell infiltration and other features [21–23]. However, the diagnosis of graft rejection would be easier if 3D imaging of the morphology and microstructures of the small bowel grafts was available [8]. Megacystis microcolon intestinal hypoperistalsis syndrome (MMIHS) is a type of gastrointestinal dysmotility syndromes [24]. The prognosis of patients with MMIHS is poor, and most patients require total parenteral nutrition (TPN), which frequently results in TPN-related liver failure, loss of venous access, or catheter-related sepsis [25]. Therefore, intestinal or multivisceral transplantation is often a valuable and life-saving therapeutic alternative [25]. MMIHS patients typically have abnormalities in the muscle layer, myenteric plexus, or the interstitial cells of Cajal (ICCs) [24, 25]. However, the histological and immunohistochemical examinations of the small intestines offer limited perspectives of these structures, and the complex relationship of the ICCs and the enteric nervous system and other surrounding structures could not be clearly delineated by the histopathological studies [26, 27]. In addition, it is difficult to use the standard 2D analysis of a few sections of the specimen to provide a comprehensive spatial view of the microstructures of the small intestine. In contrast, 3D imaging of the intestinal tissues may be a valuable tool for the evaluation and diagnosis of intestinal disorders. 

In this report, we used the 3D imaging system for the visualization of the 3D structure of the crypt organoids and the small bowels of the donors and recipients. The 3D structures of these tissues were observed and compared with conventional 2D histopathology and immunohistochemistry. 

## 2. Materials and Methods

### 2.1. Animal Experiments

The animal experiments were approved by the Committee on Laboratory Animal Research of the Far Eastern Memorial Hospital (FEMH), Taiwan, and conducted according to the guidelines of the Laboratory Animal Center of the FEMH. Five-week-old C57BL/6JNarl mice, weighing 15–20 grams, were used for the experiments. The mice were provided food and water ad libitum on a 12 : 12 h day-night cycle (lights on from 6 : 00 a.m. to 6 : 00 p.m.), with the room temperature maintained at approximately 20°C. 

### 2.2. Isolation and *In Vitro* Culture of the Stem Cell Fractions from the Intestinal Tissues of Mice

Isolation of the intestinal stem cell fractions was performed with a modification of the method that was described previously [28]. Briefly, the intestinal tissues of the mice were sliced into fragments and cut longitudinally, followed by soaking in ice-cold PBS containing gentamicin (0.5 mg/mL), with gentle shaking at 4°C for 10 min to remove feces and contaminants. The tissue fragments were then washed in ice-cold PBS (Mg^2+^/Ca^2+^) with gentle shaking at 4°C for 20 min. The fragments were next incubated in PBS0 (PBS containing 1 mM EDTA/1 mM EGTA) with gentle shaking at 4°C for 10 min. After discarding the supernatant with suspended small tissue pieces, fresh PBS0 was immediately added and mixed with the fragments by vortex. The cells that were suspended in PBS0 were collected. The process of PBS0 incubation and PBS0 vortex was repeated 7 times. Then, the cells were designated as the ISC fraction and used for *in vitro* culture. 5,000 cells of the ISC fraction were suspended in 500 *μ*L of ice-cold, laminin-rich Matrigel and were plated in a 12-well plate. After the polymerization of Matrigel, 500 *μ*L crypt culture medium (DMEM/F12 medium containing 100 ng/mL Noggin, 500 ng/mL R-spondin 1, 50 ng/mL EGF, 1 *μ*M Jag-1, 10 *μ*M Y-27632, 2 mM L-glutamine, 100 units/mL penicillin, 100 *μ*g/mL streptomycin, and 10% FBS) was added to cover the Matrigel. This covering medium was replaced every 4 days. For cell passage, the medium was discarded and the Matrigel was dissolved by a recovery solution (BD, Franklin Lakes, NJ, USA). The crypt cells were washed twice with PBS and then transferred into fresh Matrigel and crypt culture medium as above. The interval of cell passage was approximately once every 2 weeks, with 1 : 5 ratio for amplification. 

### 2.3. Specimens from Patients Receiving Small Intestinal Transplants

The study was approved by the institution review board of FEMH, Taiwan. The consent was obtained from the subjects after the nature of the procedures had been fully explained. Isolated intestinal transplantation was performed, with size- and blood type-matched deceased donors being used. The recipient's native bowels and the donor bowels were used for histopathological examination after small bowel transplantation, and a scheduled ileostomy biopsy was also performed. In addition, the intestinal tissues are subjected to 3D imaging and compared with conventional histopathological and immunohistochemical examination. 

### 2.4. Optical Clearing of Crypt Organoids or Biopsied Intestinal Tissues

FocusClear (CelExplorer, Hsinchu, Taiwan), an optical clearing agent, was used in this study. The crypt organoids were fixed with 4% paraformaldehyde for 2 h, followed by soaking in FocusClear for 2 h, according to the manufacturer's instruction. Similarly, the paraformaldehyde-fixed intestinal tissues were soaked in FocusClear overnight. After soaking, the samples were observed with an optical microscope (Zeiss Axio Imager Z2, Carl Zeiss Microimaging GmbH, Göttingen, Germany). 

### 2.5. Fluorescence Staining and Optical Clearing of Crypt Organoids and Intestinal Tissues

The fluorescence staining and optical clearing procedures were performed as previously described [26, 29]. Briefly, the fixed crypt organoids or intestinal tissues were immersed in 2% Triton X 100 solution for 2 days at 15°C for permeabilization. For fluorescence staining, propidium iodide (Invitrogen, Carlsbad, CA, USA) was used to detect the nucleus; DiD (4-chlorobenzene sulfonate salt, Molecular Probes, Eugene, OR, USA) to detect the cell membrane; the monoclonal rabbit anti-PGP 9.5 antibody (Epitomics, Burlingame, CA, USA) to reveal the neural structure; and the C-kit antibody (Santa Cruz Biotechnology, Santa Cruz, CA, USA) to demonstrate the ICCs. Before applying the antibody, the tissue was rinsed in PBS. This was followed by a blocking step, incubating the tissue with the blocking buffer (2% Triton X-100, 10% normal goat serum, and 0.02% sodium azide in PBS). The primary antibody was then diluted in the dilution buffer (1 : 50, 0.25% Triton X-100, 1% normal goat serum, and 0.02% sodium azide in PBS) to replace the blocking buffer and was incubated for 1 day at 15°C. An Alexa Fluor 647 conjugated goat anti-rabbit secondary antibody (1 : 200, Invitrogen, Carlsbad, CA, USA) was used to reveal the fluorescently labeled structures. The labeled specimens were then immersed in the FocusClear solution overnight for optical clearing before being imaged via confocal microscopy (Zeiss LSM 510 Meta confocal microscope, Carl Zeiss Microimaging GmbH, Göttingen, Germany).

### 2.6. Reconstitution of Tissues after 3D Imaging for Conventional Histopathology and Immunohistochemistry

After fluorescence staining, optical clearing was reversed by washing the tissues with saline. The specimens were subsequently processed with H & E staining or immunohistochemistry for conventional analysis comparisons.

### 2.7. Confocal Microscopy, Image Analysis, Processing, and Projection

Confocal microscopy was used to analyze the fluorescently labeled crypt organoids and intestinal tissues, as previously described [26, 29]. Briefly, the Zeiss LSM 510 Meta confocal microscope equipped with a 10x “Fluar” objective lens was used to acquire the images (optical section: 10 *μ*m; *Z*-axis increment: 5 *μ*m) (10 *μ*m interval increment along the *Z*-axis, with a 5 *μ*m overlap of previous image). The 25xLD “Plan-Apochromat” glycerine immersion lenses were used to acquire high-resolution images of the specimens (optical section: 5 *μ*m; *Z*-axis increment: 2.5 *μ*m). Each confocal micrograph consisted of 1024 (*X*) × 1024 (*Y*) pixels. The Avizo 6.2 image reconstruction software (VSG, Burlington, MA, USA) and the Zen software (Carl Zeiss, Jena, Germany) were used for analysis, processing, and projection of the confocal image stack. The Avizo software was operated under a Dell T7500 workstation (96 GB memory) with a Linux operating system.

### 2.8. Immunohistochemistry

For immunohistochemical staining, 8 *μ*m cryostat sections of the tissues were air-dried for 1 h at room temperature. Sections were fixed in acetone at 4°C for 5 min, washed with PBS, and then incubated with 3% H_2_O_2_ in methanol for 30 min. The sections were then dried and incubated with blocking solution for 30 min. The specific antibody (monoclonal rabbit anti-PGP 9.5 antibody, Epitomics; C-kit antibody, Santa Cruz Biotechnology) was diluted in 1% bovine serum albumin in PBS. The antibodies were layered onto the section and incubated at 4°C for more than 12 h. After exposure to the secondary antibody, the sections were processed with DAKO LSAB2 System HRP (DAKO Corp., Carpinteria, CA, USA) according to the manufacturer's instructions. The slides were then counterstained with hematoxylin, mounted with a coverslip, and viewed under a light microscope. 

## 3. Results

### 3.1. Optical Clearing of the Crypt Organoids and the Biopsied Intestinal Tissues

**Figure 1:**
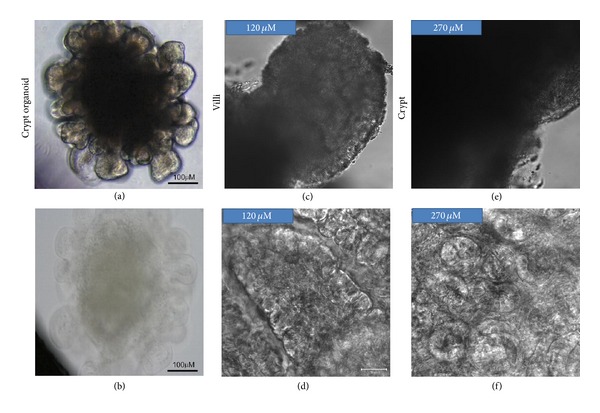
Optical clearing increased light transmission of the crypt organoids and small bowel mucosa. (a) Crypt organoid in PBS. (b) Optically cleared crypt organoid. (c) Small bowel mucosa in PBS at 120 *μ*m deep below the villus top. (d) Optically cleared small bowel mucosa at 120 *μ*m deep below the villus top. (e) Small bowel mucosa in PBS at 270 *μ*m deep below the villus top. (f) Optically cleared small bowel mucosa at 270 *μ*m deep below the villus top. Bar: 100 *μ*m.

We first tested whether FocusClear, an optical clearing agent, could increase the transparency of the crypt organoids (approximately 300 *μ*m in thickness) of intestinal stem cells from* in vitro* culture. The phosphate buffered saline (PBS)-treated crypt organoids were opaque and their central regions were dark (Figure 1(a)); in contrast, the FocusClear-treated crypt organoids (Figure 1(b)) were transparent. We also studied the effects of FocusClear on the transparency of the grafted ileum from the MMIHS patient that received a small bowel transplant (Figures 1(c)–1(f)). The PBS-treated specimens appeared dark and opaque (Figures 1(c) and 1(e)). In contrast, the specimens undergoing FocusClear treatment were transparent and the cross-sections of the specimens revealed clear pictures at different depths, with villi noted at a depth of 120 *μ*m and the crypt base at a depth of 270 *μ*m below the villus top (Figures 1(d) and 1(f)). These results demonstrated that FocusClear could provide an optical clearing effect on the prefixed crypt organoids and human small bowel specimens, which allowed for clear visualization of the villus from the top to the crypt base for approximately 300 *μ*m thickness.

### 3.2. Propidium Iodide Staining and 3D Confocal Imaging of the Crypt Organoids

**Figure 2:**
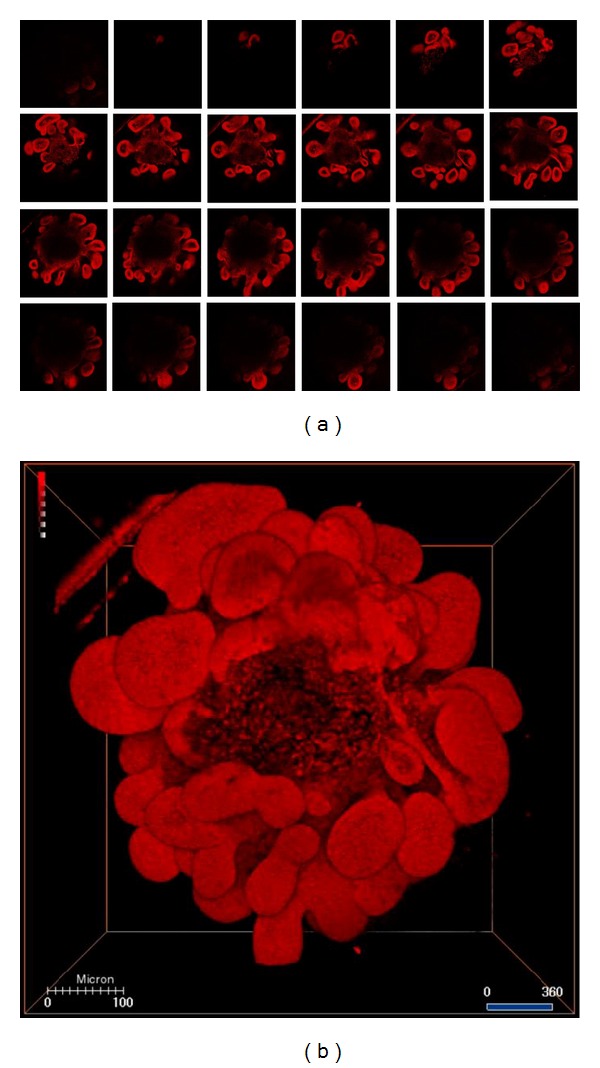
Serial confocal microscopy images and 3-dimensional reconstruction of the propidium iodide-labeled crypt organoid. (a) Serial sections from the top to the base of the crypt organoid. (b) 3D reconstruction from these serial images. Bar: 100 *μ*m.

The crypt organoids were subjected to propidium iodide (PI) staining prior to FocusClear treatment. By 2D images for PI-staining, the red fluorescent signals throughout the crypt organoid were clear, and the shape of the crypt organoid and the protruded villi-crypt domains were well-defined (Figure 2(a)). After reconstruction of the 2D serial optical images, the 3D structures of the crypt organoids were observed as a spherical body surrounded by protruded villi-crypt domains (see Figure 2(b) and Video S1 in the Supplementary Material available online at http://dx.doi.org/10.1155/2013/624342). 

### 3.3. Propidium Iodide Staining and 3D Confocal Imaging of the Biopsied Intestinal Tissues with Acute Rejection

**Figure 3:**
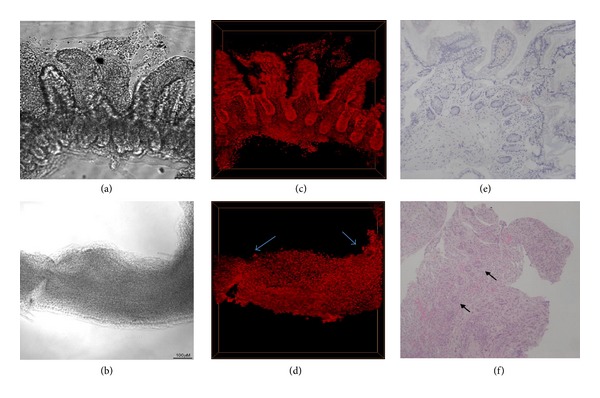
Propidium iodide staining and 3D confocal imaging of the biopsied intestinal tissues with acute rejection. ((a) and (b)) Optically cleared intestinal tissues without (a) or with (b) acute rejection. ((c) and (d)) Propidium iodide fluorescence signals of the intestinal tissues without (c) or with (d) acute rejection. ((e) and (f)) Histopathology of the intestinal tissues without (e) or with (f) acute rejection (H and E stain, 100x). Bar: 100 *μ*m. Arrow indicated residual crypts in the intestinal tissues with acute rejection.

To observe the structures of the grafted ileum with or without acute rejection after small bowel transplantation, the permeabilized small bowel specimens were stained with PI prior to FocusClear treatment (Figure 3). Figures 3(a) and 3(b) show that FocusClear increased the transparency of intestinal tissues. The 3D structures of the PI-labeled intestinal tissues without rejection could be clearly seen from the villus top down to the crypt base (Figure 3(c) and Videos S2 and S3), which were consistent with the normal intestinal features in histopathology (Figure 3(e)). In contrast, the PI-labeled intestinal tissues with rejection showed few crypts and absence of villi in the 3D images (Figure 3(d) and Videos S4 and S5). The histopathology of the intestinal tissues with rejection showed denuded and ulcerated mucosa, loss of crypts and mononuclear cell infiltration (Figure 3(f)), which were consistent with the 3D images. 

### 3.4. Reconstitution of Fluorescently Stained Intestinal Tissues for Histopathology

**Figure 4:**
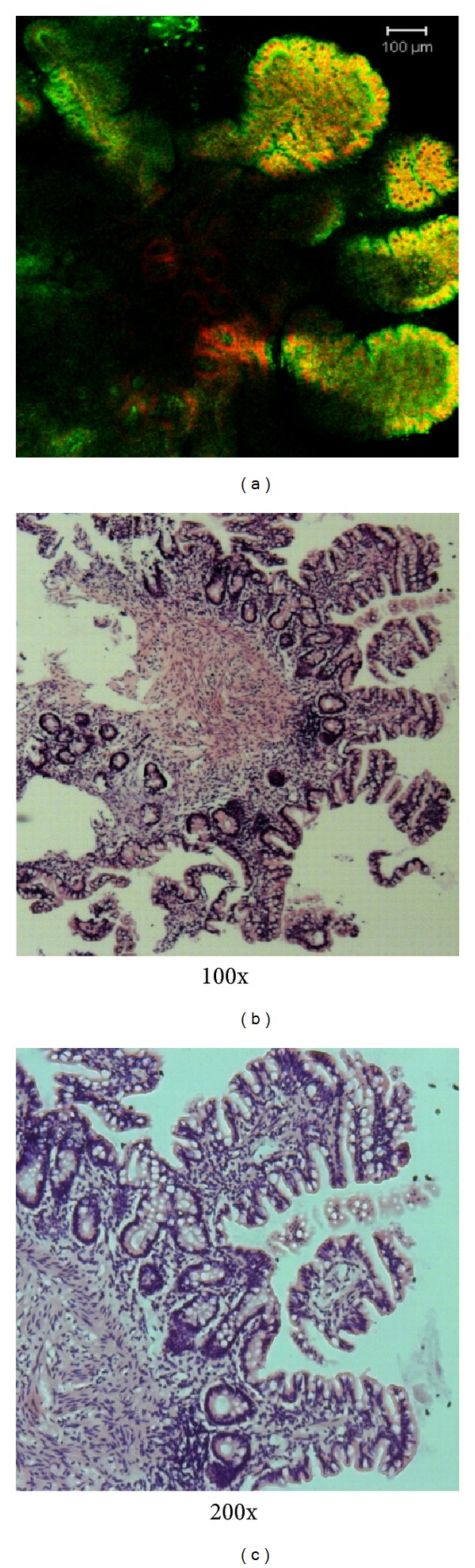
Reconstitution of fluorescence-stained intestinal tissues for histopathology. (a) The 3D image of the intestinal mucosa section was shown by the fluorescence staining of propidium iodide (red) for the nucleus and DiD (green) for the cell membrane. Bar: 100 *μ*m. ((b) and (c)) Histology of the intestinal tissues reconstituted from fluorescence staining (H & E staining, (b) 100x, (c) 200x ).

We compared the results of the 3D imaging of the intestinal tissues and the histopathology of the same specimen reconstituted from the 3D imaging. The cross-section of the intestinal mucosa was well defined by PI staining for the nucleus and 4-chlorobenzene sulfonate salt (DiD) staining for the cell membrane (Figure 4(a)). Furthermore, the histopathology of the tissues used after fluorescence staining displayed intact intestinal mucosa with well-arranged villi (Figures 4(b) and 4(c)). In addition, the 3D image and the histopathology showed similar results. 

### 3.5. PGP9.5 Staining of the Myenteric Plexus and Nerve Fibers in Donor and Recipient Intestinal Tissues from MMIHS Patient That Received Intestinal Transplant

**Figure 5:**
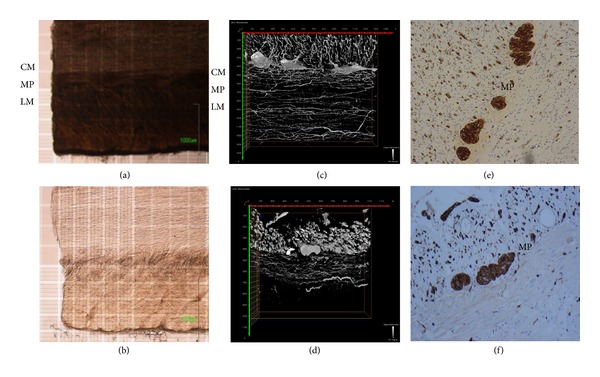
PGP9.5 staining of the myenteric plexus and nerve fibers in the donor and recipient intestinal tissues from a MMIHS patient that received intestinal transplantation. (a) Intestinal tissues in PBS. (b) Optically cleared intestinal tissues. ((c) and (d)) The permeabilized donor (c) and recipient (d) intestinal tissues were stained with PGP9.5 prior to FocusClear treatment. ((e) and (f)) PGP9.5 immunohistochemical studies of the intestinal tissues ((e), donor; (f), recipient, 100x). CM: circular muscle layer; MP: myenteric plexus; LM: longitudinal muscle layer. Bar: 100 *μ*m.

A comparison of the donor and recipient intestinal tissues from patient with MMIHS that received an intestinal transplant was performed by using 3D images (Figure 5). In the PBS-treated intestinal tissues, the muscle layers appeared opaque (Figure 5(a)); in contrast, after immersion in FocusClear, the intestinal tissues, including the longitudinal and circular muscles, became transparent (Figure 5(b)). PGP9.5 was used to stain the myenteric plexus and nerve fibers in the muscle layers. In the 3D images, the myenteric plexus was at the interspace between longitudinal and circular muscles. In the donor's intestine (Figure 5(c)), the muscle layers were thick; the nerve network was well organized, with compact bundles present in the muscle layer; and the nerve fibers were prominent, well defined, and elongated to form a complex network. In contrast, in the recipient's intestine (Figure 5(d)), the muscle layers were thin; the nerve network was irregular and distorted; the nerve fibers were crowded, small in diameter, and short, with blunted ends. Interestingly, the myenteric plexus showed no difference between the donor and recipient intestinal tissues. The PGP9.5 immunohistochemical studies of the intestinal tissues (Figure 5(e): donor; Figure 5(f): recipient) showed similar results compared to the 3D images. 

### 3.6. C-Kit Staining of Interstitial Cells of Cajal in the Donor and Recipient Intestinal Tissues from MMIHS Patient that Received Intestinal Transplant

**Figure 6:**
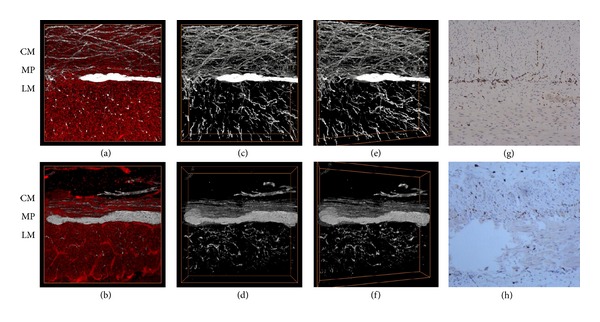
C-kit staining of interstitial cells of Cajal in the donor and recipient intestinal tissues from a MMIHS patient that received intestinal transplantation. ((a) and (b)) Propidium iodide and C-kit staining in the donor (a) and recipient (b) intestinal tissues. ((c) and (d)) C-kit staining in the donor (c) and recipient (d) intestinal tissues. ((e) and (f)) C-kit staining in the donor (e) and recipient (f) intestinal tissues, 15 degree-oblique images of ((c) and (d)). ((g) and (h)) C-kit immunohistochemical studies of the intestinal tissues reconstituted from fluorescence staining. ((g), donor; (h), recipient, 100x). CM: circular muscle layer; MP: myenteric plexus; LM: longitudinal muscle layer. Bar: 100 *μ*m.

The distribution of the ICCs in the donor and recipient intestinal tissues from MMIHS patient that received intestinal transplant was studied with the 3D imaging system (Figure 6). As illustrated in the 3D projection of donor intestine, the muscles were labeled by PI stain and the C-kit-positive signals were mainly distributed in the circular muscle layer and around the myenteric plexus (Figures 6(a), 6(c), 6(e), and 6(g)). In contrast, in the recipient intestine, there were fewer ICCs compared to the donor intestinal tissues, in both the circular and longitudinal muscle layers and the area around the myenteric plexus (Figures 6(b), 6(d), 6(f), and 6(h)). 15 degree-oblique 3D images (Figures 6(e) and 6(f)) provided a clearer depiction of the spatial distribution and orientation of the ICCs. The C-kit immunohistochemical studies of the recipient intestinal tissues revealed decreased ICCs compared to the donor tissues (Figures 6(g) and 6(h)), and the immunohistochemical features mirrored the results that were observed with the 3D images.

## 4. Discussion

The regeneration activity of intestinal stem cells plays an important role in the recovery of the intestinal mucosa after various physical and pathological injuries [28, 30, 31]. Furthermore, ISCs may form the neointestinal mucosa in various tissues [14, 15, 17, 20]. In the long-term culture system, ISCs could undergo multiple fission events to generate crypt organoids, which contain all differentiated cell types of the villi [16, 18, 19]. However, during these culture studies, only the 2D morphology could be observed. In this study, we applied the 3D imaging method to visualize the structures of the crypt organoids from the ISC *in vitro* culture. Tissues were processed by an optical clearing agent such as FocusClear to allow for optical imaging, laser penetration, and fluorescence detection by confocal microscopy [8]. We found that the crypt organoids became transparent after FocusClear treatment. In addition, the fluorescence-labeled specimens that were optically cleared by FocusClear could be directly visualized by confocal microscope. The serial optical sections of the crypt organoids showed well-delineated images, which could be reconstructed into a 3D image, and the spatial relationship between the labeled cells and surrounding tissues could be identified. From this method, we achieved an integrated visualization of the 3D structure of the crypt organoids in detail. These results demonstrated that the process of optical clearing could provide high-definition transparent images of the microstructures and that the 3D imaging approach could be applied to 300 um thick tissues. 

Intestinal transplantation is widely used for patients with irreversible intestinal and total parenteral nutrition failure [32]. Because intestinal transplant recipients may be asymptomatic during the first three posttransplant months but may experience low-grade histological rejection, these patients often fare more poorly than those lacking early histological rejection [23, 33]. Therefore, surveillance endoscopic biopsies of the small bowel transplant are performed regularly, following transplantation, to prevent allograft rejection [23]. Although the conventional 2D histopathological examination of the small bowel sections is essential for the detection of rejection, it only offers the visualization of the intestinal structures at a specific cut plane and these data may produce diagnostic errors, due to artifacts, incomplete sampling, or poor orientation of embedded specimens [6, 8]. In contrast, 3D imaging shows the gross appearance and microstructures of the small intestine and could provide a more comprehensive view of the mucosal changes, patterns of the villi and crypts, the nerve fiber network, muscle structures, and vasculature [8]. Therefore, to monitor the recovery after transplantation and detect possible rejection, the integration of 3D imaging and the conventional 2D histology might provide a new way to visualize the microstructure and tissue network of the grafted intestine. In our study, we found that the transparent tissues derived from optical clearing allowed penetrative confocal microscopy for in-depth presentation of the 3D tissue structures, which could not be easily portrayed by the 2D analysis. Furthermore, the intestinal tissues with rejection showed few crypts and absence of villi in the 3D images, which were also consistent with the histopathology results, showing denuded and ulcerated mucosa, loss of crypts, and mononuclear cell infiltration. In addition, the tissue specimens that were subjected to the 3D imaging process could be reconstituted for regular histopathology and immunohistochemistry, so that the same tissues could be used for both conventional histopathology/immunohistochemistry and 3D imaging, and these data could be compared. By combining these methods we could achieve a comprehensive view of the intestinal tissues, which may ultimately help the early diagnosis of graft rejection after intestinal transplantation. 

Histologically, most MMIHS patients have normal ganglion cells in the myenteric and submucosal plexuses; however, there have been case reports of decreased ganglia in some patients and hyperganglionosis and giant ganglia in others [25]. In addition, MMIHS had been reported to be associated with the deficiency of ICCs networks [27]. Therefore, in this study, the 3D imaging system was further applied to investigate the enteric nervous system of the intestinal tissues from MMIHS patient that received intestinal transplantation. From the 3D imaging, ICCs form a dense network around the myenteric plexus and within the muscle layers in the donor intestinal tissues; in contrast, only few single ICCs are detected in the recipient intestinal tissues. These data were consistent with the histopathology of MMIHS patients in the literature [34]. However, this 3D imaging system provided detailed, global representations of the anatomical and pathological changes of the enteric nervous system in the MMIHS patient, whereas the 2D histology provides a limited perspective of the enteric nervous system due to the dispersed nature of nerve fibers. Thus, the integration of the 3D imaging method could compensate the inherent defect of the 2D histological analysis about the study of the enteric nervous system in patients with motility disorders. 

In summary, this study utilized the 3D imaging system to aid in the visualization of the 3D structures of the crypt organoids and the biopsied intestinal tissues from patient with acute rejection after intestinal transplant and from MMIHS patient receiving intestinal transplantation. The 3D imaging provided clear spatial views of the crypt organoids from the *in vitro* stem cell cultures and revealed 3D pictures of the graft rejection after small bowel transplantation and the global features of the intestinal structures including the enteric nervous system. In addition, the 3D imaging specimen preparation protocol was compatible with the routine histological and immunohistochemical processing procedure, and the specimens could be reconstituted so that conventional histopathology could be performed. Thus, the integration of 3D imaging with the 2D histopathology may provide an alternative approach to the study of small bowel pathology; certainly, more studies are necessary before the 3D imaging system can be applied clinically. 

## Supplementary Material

Video S1. 360-degree presentation of the propidium iodide-labeled crypt organoid as in Figure 2B. This video provided panoramic view of the 3D crypt organoid. Dimensions of the projected volume: 512 **μ**m (X) × 512 **μ**m (Y) × 150 **μ**m (Z, depth).
Video S2 and S3. 360-degree presentation of the propidium iodide-labeled biopsied intestinal tissues (without rejection) after intestinal transplantation, as in Figure 3C. This video provided panoramic view of the 3D intestinal tissue. Dimensions of the projected volume: 921 **μ**m (X) × 912 **μ**m (Y) × 150 **μ**m (Z, depth).
Video S4 and S5. 360-degree presentation of the PI-labeled biopsied intestinal tissues (with acute rejection) after intestinal transplantation, as in Figure 3D. This video provided panoramic view of the 3D intestinal tissues with rejection and arrows indicated residual crypts. Dimensions of the projected volume: 1024 **μ**m (X) × 1024 **μ**m (Y) × 100 **μ**m (Z, depth).
